# Comprehensive genomic profiling in routine clinical practice leads to a low rate of benefit from genotype-directed therapy

**DOI:** 10.1186/s12885-017-3587-8

**Published:** 2017-08-30

**Authors:** Talal Hilal, Mary Nakazawa, Jacob Hodskins, John L. Villano, Aju Mathew, Guarav Goel, Lars Wagner, Susanne M. Arnold, Philip DeSimone, Lowell B. Anthony, Peter J. Hosein

**Affiliations:** 1 Division of Hematology and Medical Oncology, Mayo Clinic, Phoenix, AZ USA; 2 University of Kentucky College of Medicine, Lexington, KY USA; 3 Division of Medical Oncology, University of Kentucky, Markey Cancer Center, Lexington, KY USA; 4 Department of Medicine, Division of Hematology/Oncology, University of Pittsburgh Cancer Institute, Pittsburgh, PA USA; 5 Hematology/Oncology, University of Miami School of Medicine and Sylvester Comprehensive Cancer Center, Miami, FL USA

**Keywords:** Genotype-directed therapy, Profiling, Genomics, Cancer therapeutics

## Abstract

**Background:**

Describe a single-center real-world experience with comprehensive genomic profiling (CGP) to identify genotype directed therapy (GDT) options for patients with malignancies refractory to standard treatment options.

**Methods:**

Patients who had CGP by a CLIA-certified laboratory between November 2012 and December 2015 were included. The medical records were analyzed retrospectively after Institutional Review Board (IRB) approval. The treating oncologist made the decision to obtain the assay to provide potential therapeutic options. The objectives of this study were to determine the proportion of patients who benefited from GDT, and to identify barriers to receiving GDT.

**Results:**

A total of 125 pediatric and adult patients with a histologically confirmed diagnosis of malignancy were included. Among these, 106 samples were from adult patients, and 19 samples were from pediatric patients. The median age was 54 years for adults. The majority had stage IV malignancy (53%) and were pretreated with 2–3 lines of therapy (45%). The median age was 8 years for pediatric patients. The majority had brain tumors (47%) and had received none or 1 line of therapy (58%) when the profiling was requested. A total of 111 (92%) patients had genomic alterations and were candidates for GDT either via on/off-label use or a clinical trial (phase 1 through 3). Fifteen patients (12%) received GDT based on these results including two patients who were referred for genomically matched phase 1 clinical trials. Three patients (2%) derived benefit from their GDT that ranged from 2 to 6 months of stable disease.

**Conclusions:**

CGP revealed potential treatment options in the majority of patients profiled. However, multiple barriers to therapy were identified, and only a small minority of the patients derived benefit from GDT.

## Background

Carcinogenesis is a multi-step process propelled by genomic alterations that leads to dysregulation of signaling pathways, which consequently gives rise to qualities that enable tumor proliferation and dissemination [1]. Our understanding of the molecular processes underlying malignancies has translated to targeted therapies, which have transformed the clinical management of some cancers. Indeed, the landscape of systemic therapy in certain malignancies is evolving from its dependence on nonselective cytotoxic therapies to one that includes the utilization of selective inhibitors [2]. Since the first breakthrough in molecular targeted therapy with imatinib, whose action against BCR-ABL kinase produced robust responses in chronic myeloid leukemia (CML) [3], various oncogenic drivers of the proliferative phenotype have been uncovered and translated into therapies. The use of trastuzumab, a humanized monoclonal antibody against human epidermal growth factor receptor 2 (HER2) is standard of care for HER2 overexpressed breast cancers [4] and more recently approved for HER2 overexpressed gastric cancer, which represents the first targeted therapy in this malignancy [5]. While these well-studied and validated alterations are routinely targeted in clinical practice, patients with other alterations in frequently mutated pathways may also benefit from targeted therapies.

Today, comprehensive genomic profiling (CGP) of tumors can provide insight into clinically relevant genetic alterations (CRGAs), with goals of guiding clinical decision-making and augmenting therapeutic options. The accessibility of this technology facilitates the shift towards precision medicine by identifying specific patient populations that are most likely to derive benefit from a particular therapy. However, the proportion of patients that end up receiving genotype-directed therapy (GDT) represents the minority of total patients profiled, despite a large proportion having actionable genetic alterations [6]. In addition, only a small fraction of patients profiled in such studies derive benefit from the treatment [7]. The reasons for the marked divergence in patients profiled to have actionable mutations and those that ultimately receive GDT have not been systematically studied.

Herein we present a single institution study of the outcomes of CGP in the clinical management for these patients, with focus on exploring the reasons for which patients with actionable mutations did not receive GDT.

## Methods

### Study population/design

This was a retrospective, single-center, observational study that reviewed the medical records of adult (> or equal to 18 years of age) and pediatric (<18 years of age) patients with a histologically confirmed diagnosis of malignancy. All patients who had CGP between November 2012 and December 2015 at the University of Kentucky were included. Data cut-off was in June 2016. No restrictions on tumor histology, disease stage, subsequent or previous treatment, or performance status were imposed. Malignant tumors were tested with a commercially available CGP assay performed by Foundation Medicine. The decision to obtain the assay for a particular patient was at the discretion of the primary physician. Archived tissue from patients’ diagnostic biopsies or surgical resection was used for testing. The Institutional Review Board (IRB) of the University of Kentucky approved the study.

### Genomic testing

The methodology used in next-generation sequencing has been well-described in previous publications and replicated in the majority of commercial CGP platforms [8]. The Foundation Medicine platform simultaneously sequences the coding region of 236 cancer-related genes plus introns from 19 genes often rearranged or altered in cancer to a typical median depth of coverage of greater than 250X. Sample requirements are ≥40 μm tissue, of which a minimum of 20% is of malignant origin, on 8 to 10 unstained slides or in a formalin-fixed paraffin-embedded block. The sensitivity of the test is reported at >99% for base substitutions, >97% for indels, >95% for copy number alterations, and >90% for rearrangements. The specificity is reported at >99% for all classes of genomic alterations [9].

### Report interpretation

The Foundation Medicine report provided a list of CRGAs with suggested treatment options that were on-label, off-label, or both. In addition, a list of phase 1–3 trials that may be recruiting for patients with specific CRGAs was provided. The treating oncologist made his/her own interpretations to identify treatments based on the report. Patients with CRGAs with on-label options have either received the recommended, on-label therapy, or will be receiving it at which point the report was saved for potential future use. Patients with CRGAs without on-label options were either referred for a clinical trial that was selected by the treating oncologist, or received an off-label therapy that was suggested by the report. If the options listed in the report were deemed unlikely to be effective by the treating oncologist, an alternative therapy was used.

### Clinical endpoints

The primary objective was to identify the barriers to receiving GDT. The secondary objectives were to determine the percentage of patients with CRGAs, and the proportion of patients who benefitted from GDT either by having a response to therapy defined by RECIST v.1.1 criteria or disease stabilization. Patients were considered to have received GDT only when the test result identified genetic alterations to which an off-label therapy or a clinical trial could be offered. Patients with a previously established genomic alteration discovered on routine clinical testing for which a standard of care targeted agent was available (e.g. trastuzumab for HER2 positive breast cancer) were not counted to have received GDT based on the results of the test.

## Results

### Patient characteristics

Baseline demographics are displayed in Table 1. The median age was 54 years for adults, with equal gender distribution. The majority had stage IV malignancy (53%) followed by stage III (25%). Most patients were pretreated with 2–3 lines of therapy (45%); 38% received one or no prior lines of therapy. The median age was 8 years for pediatric patients, with equal gender distribution. The majority had either brain tumors (47%) or stage IV malignancy (32%). Most patients had received none or 1 line of therapy (58%) when the profiling was requested. Median follow-up was 12.9 months (range, 5.6–43.3 months).

**Table 1:** Baseline characteristics and demographic information

Variable		Adult cohort *n* = 106	Pediatric cohort *n* = 19
Age at diagnosis (years)	Median	54	8
	Range	21–93	2–17
Sex	Female	54 (51%)	9 (47%)
	Male	52 (49%)	10 (53%)
Stage (TNM)	I	7 (7%)	4 (21%)
	II	13 (12%)	-
	III	26 (25%)	-
	IV	56 (53%)	6 (32%)
	Other (Non-TNM)	-	9 (47%)
	Unknown	4 (3%)	-
Prior lines of systemic therapies	0–1	41 (38%)	11 (58%)
	2–3	48 (45%)	5 (26%)
	4 or more	18 (17%)	3 (16%)
Prior standard-of-care targeted therapy		11 (10%)	-

One hundred and twenty-five patient tumor samples were submitted for testing. Among these, 106 tumor samples were from adult patients, and 19 tumor samples were from pediatric patients (Table 2). In adults, the most common tumor histology evaluated were sarcomas (*n* = 24), followed by non-small cell lung cancer (NSCLC) (*n* = 17), and breast and colorectal (*n* = 11 each). In pediatric patients, the most common tumors evaluated were primary brain tumors (*n* = 9).

**Table 2:** Histologies of tumors profiled in adult and pediatric patients

Adult (*n* = 106)	
Head and Neck	
Salivary gland	1
HNSCCa	2
Thyroid	2
Esthesioneuroblastoma	1
Lung	
NSCLC	17
SCLC	1
Neuroendocrine	1
Breast	11
Esophageal	2
Gastric	3
Hepatobiliary	
Hepatocellular carcinoma	1
Cholangiocarcinoma	8
Gallbladder Carcinoma	2
Pancreatic adenocarcinoma	4
Colorectal	11
Neuroendocrine - GI	2
Adrenocortical carcinoma	3
Urothelial/Bladder Carcinoma	1
Ovarian carcinoma	1
Uterus carcinoma	1
Neuroendocrine - GU	1
Melanoma	1
Sarcoma	
Osteosarcoma	3
GIST	1
Leiomyosarcoma	2
Liposarcoma	3
NOS/Other	15
Unknown primary	5
TOTAL	106
Pediatric (*n* = 19)	
Brain tumors	
Juvenile pilocytic astrocytoma	1
Oligodendroglioma	1
Ependymoma	2
Anaplastic astrocytoma	2
GBM	1
Atypical teratoid/rhabdoid	2
Neuroblastoma	2
Sarcoma	
Giant cell tumor	1
Osteosarcoma	1
Rhabdomyosarcoma	1
Ewing	3
NOS/Other	1
Melanoma	1
TOTAL	19

### Clinically relevant genetic alterations

Four patients (2 adults and 2 pediatric patients) had insufficient tumor DNA in the specimens submitted to perform profiling. In adults, at least one genomic alteration was identified in 97 tumor samples (93%) all of which had phase 1 clinical trial options and were therefore defined as CRGAs. Twenty-five adult patients (24%) had genomic alterations for which an on-label, FDA-approved option was available – most of these alterations were already known prior to CGP because of standard-of-care biomarker testing (e.g. RAS mutation testing in colorectal cancer). Seventy-eight adult patients (74%) had genomic alterations for which an off-label option was available. In pediatric patients, at least one genomic alteration was identified in 14 tumor samples (82%) all of which had phase 1 clinical trial options. There were no pediatric patients with genomic alterations for which an on-label, FDA-approved option was available, but there were 11 (65%) with a genomic alteration for which an off-label option was available (see Table 3).

**Table 3:** Results of profiling

Variable		Adult cohort *n* = 106	Pediatric cohort *n* = 19
Valid results		104 (98%)	17 (90%)
		Adult cohort *n* = 104	Pediatric cohort *n* = 17
On-label options only		3 (3%)	0
Off-label options only		55 (53%)	11 (65%)
Both on- and off- label options		22 (21%)	0
Clinical Trial options	*Phase I*	96 (92%)	14 (82%)
	*Phase II*	76 (73%)	11 (65%)
	*Phase III*	11 (11%)	0

**Fig. 1:**
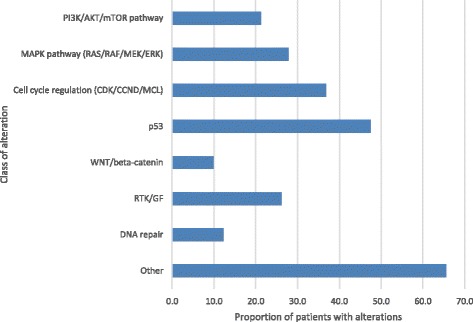
Proportion of samples with alterations by class of molecular pathway

Genomic alterations were detected across a range of functionally relevant molecular pathways. Cell cycle regulation genes were the most frequently altered pathways with mutations, amplifications or deletions present in 37% of tumors. A mutation in the p53 gene was the most frequently identified single gene alteration, found in 48% of tested samples. Alterations in the phosphatidylinositol 3-kinase (PI3K)-AKT pathway and the mitogen-activated protein kinase (MAPK) pathway were identified in 21% and 28% of samples, respectively (Fig. 1).

### Treatment options and response

Out of the total of 121 patients with valid CGP results, 111 (92%) had CRGAs and were candidates for GDT either via on/off-label use or a clinical trial (phase 1 through 3). Fifteen patients (12%) received GDT based on these results including two patients who were referred for genomically-matched phase 1 clinical trials. Three patients (2%) derived benefit from their GDT that ranged from 2 to 6 months of stable disease; two patients in the adult population with lung adenocarcinoma and gastric cancer, and one of patient in the pediatric population with anaplastic astrocytoma (Table 4). Out of the total of 62 patients with stage IV malignancy, this meant 24% received GDT, and 5% derived benefit.

**Table 4:** GDT utilized based on NGS results (*n* = 15)

Patient	Tumor Type	Targeted Genetic Alterations	Therapy	Duration of benefit (months)	Best Response after GDT
1	Cholangiocarcinoma	BRAFV471F, EGFR T790 M	Sorafenib	0	PD
2	Breast cancer, recurrence	AKT3 amplification, PIK3R1 F456_E458del, PTEN loss exon 3	Everolimus	0	PD
3	NSCLC, squamous with sarcomatous features	KRAS G13D	Trametinib	0	PD
4	Head and neck squamous cell carcinoma	EGFR amplification	Cetuximab	0	PD
5	NSCLC, adenocarcinoma	NRAS Q61K	Trametinib	5	SD
6	Breast cancer	PIK3R1 K448_Y452del	Everolimus	0	PD
7	Osteosarcoma	CCND3 amplification, CDK4 amplification	Palbociclib	0	PD
8	Anaplastic astrocytoma	BRAFV600E (HGF amplification)	Vemurafenib	6	SD
9	Large cell neuroendocrine carcinoma	PTEN N323 fs*2	Everolimus	0	PD
10	Cholangiocarcinoma	IDH2 R172K	AG-881 (IDH inhibitor)	0	PD
11	Esophageal carcinoma	PIK3CA R88Q, STK11 loss	Everolimus	0	PD
12	Gastric adenocarcinoma	FLT3 amplification	Sorafenib	2	SD
13	NSCLC, squamous	HGF amplification	Crizotinib	0	PD
14	GE junction adenocarcinoma	VEGFA amplification	Sorafenib	0	PD
15	Adrenal cortical cancer	TP53 H179R	AZD1775 (WEE1 kinase inhibitor)	0	PD

### Barriers to genotype-directed therapy

Most patients were not offered GDT based on the assay report (Table 5). The most common reasons were ongoing treatment with standard-of-care therapy, or off-label therapy/clinical trial participation not suggested by assay report (*n* = 42, 40%). In other words, testing in this population was done early in the disease treatment course and the results were not applicable at the time the results were available, or the results deemed unlikely to be effective by the treating oncologist leading to off-label therapy or clinical trial participation which would have taken place regardless of whether CGP was ordered. Less common reasons were deteriorating/poor performance status (*n* = 25, 23%), and disease in remission with no indication for therapy (*n* = 18, 17%). Testing in this population was done either too late in the treatment course to be of clinical utility, or done on patients with early stage cancers who underwent curative intent therapy, respectively. Other reasons for not receiving GDT included no actionable mutations (*n* = 11, 10%), or rapid disease progression (*n* = 5, 5%).

**Table 5:** Reasons for not receiving GDT (*n* = 106)

Reason	Frequency (%)
No actionable mutations	11 (10%)
Disease in remission/no indication for therapy	18 (17%)
Patient still receiving standard of care/off-label option or clinical trial not suggested by F1 report	42 (40%)
Patient is no longer a candidate for therapy due to deteriorating or poor performance status	25 (23%)
Physician preference for no GDT specifically because of rapid disease	5 (5%)
On/off label GDT recommended or clinical trial available locally but patient declined	3 (3%)
Patient offered clinical trial but unable to travel/insurance decline	2 (2%)

## Discussion

The concept of individualizing a patient’s treatment based on a specific alteration in their tumor is attractive to oncologists and patients alike. This approach is already validated in a number of malignancies and there are now many FDA-approved therapies that require testing for a predictive biomarker [10, 11]. In these cases, where there is a validated drug-biomarker pair, the outcome with biomarker-guided therapy is usually very rewarding, leading to improvement in survival. The success of this approach in some situations has generated tremendous enthusiasm for splitting tumors into groups based on driver alterations and treating based on these results. Testing for driver genomic alterations has become routine clinical practice with the proliferation of a number of commercially available platforms for analyzing patients’ tumor specimens in an attempt to uncover vulnerabilities in the tumor that may be susceptible to new targeted therapies. However, based on the evidence we present here, as well as many other studies, it appears that the widespread uptake of genomic profiling is ahead of the evidence supporting its benefit.

The rate of benefit from GDT in our study, defined as stable disease or a partial response to therapy was 5% in patients with stage IV malignancies. This low proportion is in the range of what has been reported in other series. Table 6 shows a summary of similar real-world single-center experience with GDT in routine clinical practice. These studies all showed a high rate of potentially actionable alterations but a low rate of patients receiving matched therapy and an even lower rate of patients who derive benefit from this process [7, 12–17]. If one uses all patients entering the testing algorithm who are hoping for a “home-run” targeted therapy as the denominator, the proportion of patients who do achieve some disease control from therapy is between 2 to 8% across multiple studies.

**Table 6:** Single institution studies examining genotype-directed therapy

Study	Number of Subjects	Design	Rate of Actionable Alterations	Rate of Matched Therapy	Rate of Benefit^a^
Current study	126	Retrospective, single institution	92%	12%	2%
Vanderbilt [12]	103	Retrospective, single institution	83%	21%	8%
First MDACC [13]	1144	Prospective, phase 1 study	40%	18%	5%
UCSD [14]	34	Prospective, single institution, molecular tumor board	94%	35%	21%
Cornell [7]	97	Prospective, single institution	94%	5%	2%
Rutgers [15]	92	Prospective, single institution, molecular tumor board	96%	35%	NR
Second MDACC^b^ [16]	339	Prospective, single institution	94%	32%	NR
University of Michigan [17]	500	NR	72%	5–11%	NR

*NR* not reported

^a^Benefit defined as partial response (PR) + stable disease (SD)

^b^Supported by a grant from Foundation Medicine

Apart from the institutional series described above, the prospective SHIVA trial is a study that is often cited as an example of the limitations of GDT [18]. This study randomized patients who had one of a defined set of alterations to received genomically matched targeted therapy versus investigator’s choice chemotherapy. There was no difference in progression-free survival in the patients who received matched therapy versus those who received standard chemotherapy. Despite this apparent negative result, this study was by no means definitive and the main limitation was that the targeted drugs used were not validated against the purported targets and in a heavily pretreated population; the weak drug-target pairs were unlikely to succeed [19].

Contrast this study to the study of erlotinib versus chemotherapy for the first-line treatment of patients with lung cancer harboring an EGFR activating mutation. This study showed remarkable superiority of erlotinib in this setting with a hazard ratio for survival of 0.16 (95% CI 0.1 to 0.26) [20]. This clearly illustrates the principle that a validated drug-biomarker pair can lead to excellent outcomes. It also highlights the inherent complexity of tumors and the wide variety of genomic alterations that introduce bias in treatment selection. Many cancers do not have clear driver mutations, and targeting a random genomic alteration will have no effect of the natural history of the disease.

More recently, the ProfiLER trial, a multi-institutional prospective study from France, has reported data on 1826 patients. Approximately half (51%) had at least one actionable mutation, and 35% were deemed to be eligible for matched therapy when utilizing a molecular tumor board. Among those, 6% initiated a recommended matched therapy. The rate of benefit, which included complete response, partial response, and stable disease, was 2.4% of the total population (44% of those who initiated therapy) [21].

Our study has a number of important limitations. This was not a prospective study and the implementation of GDT was physician-dependent and not standardized. Many institutions now have molecular tumor boards that systematically review profiling results and attempt to match patients to clinical trials or the best available treatment based on the results. Even in a supervised setting like this, the rate of matched therapy is still low. The benefit derived from matched therapy, however, may be higher as shown in one single-center experience [14]. This is likely due to a more scrutinized approach in interpreting the genomic data, and selecting the appropriate patients whose tumors have shown a more indolent biology (e.g. most patients in the ProfiLER study who initiated GDT had gynecologic, colorectal and breast malignancies; many known to behave in indolent manner).

Another weakness of our study was the inconsistency in the timing of sending CGP. In some cases, testing was requested early in the disease course and the results were available while patients were still on standard-of-care therapy or in remission. Presumably the testing results could be retrieved and acted upon if the patients developed disease progression. However, the molecular alterations at the time of progression may evolve and the results of prior testing may no longer be reliable [22]. On the other hand, many patients had testing done very late in their disease course and did not have the time to have the results implemented. In general, we found that patients who had rapid disease progression or poor performance status due to their malignancy (28% of our cohort) did not have the opportunity to benefit from the results of GDT and testing these patients did not alter the outcome.

Lack of easy access to phase 1 clinical trials was also a major barrier to GDT in our study – our center had few available phase 1 trials at the time of this study. This led to many patients receiving off-label therapy that was felt to be efficacious at the discretion of the treating oncologist. Community oncology practices alike generally have limited access to phase 1 therapeutic trials making our experience reflective of the wider oncology practice. However, the use of “off-label” GDT should not be the norm since the activity of a drug targeting a particular mutation is different for distinct tumor types (e.g. BRAF inhibitors are very efficacious in BRAFV600E mutated melanoma, but lack the same activity in BRAFV600E mutated colon cancer).

So how do we move forward? Based on the evidence we provide in our study and the other studies cited here, a haphazard approach to CGP is very unlikely to help patients. Tumor profiling should be done in a deliberate way with systematic analysis of the results to guide patients into appropriate clinical trials, avoiding those who are unlikely to benefit, such as patients with rapid disease progression or those in whom standard therapy is being successfully applied. This means that the optimal time for requesting CGP may differ with distinct histologically defined tumor types. The aspect of biologic complexity of tumors and intratumor heterogeneity should also be considered, as these patients are unlikely to benefit from a targeted approach unless the target is a driver alteration. Moreover, the financial burden of CGP on both the healthcare economy and individual patients should be kept in mind. In one study of 209 patients in a community practice, the total cost was reported at $1.21 million, and 17% of patients were responsible for the full cost after exhausting financial coverage and support options [23].

The ongoing NCI-MATCH trial is an example of a basket trial that is meticulously approaching this problem. The drug-biomarker pairs have passed a minimum bar of validation and this study will provide further evidence as to whether some of the pairs will in fact benefit patients. The ASCO-TAPUR trial is another example that utilizes a molecular tumor board composed on a group of experts convened by ASCO that provide an informed decision regarding the proposed treatment. On the other hand, SPECTAcolor (Screening Patients for Efficient Clinical Trial Access in advanced colorectal cancer) is an example of a large European collaboration that aims to screen patients with colorectal cancer to improve access to molecularly defined clinical trials [24].

## Conclusions

In this study, the routine use of CGP in clinical practice was associated with minimal benefit to patients in terms of disease control. Furthermore, we were able to identify various barriers to implementation of GDT that should be taken into consideration before requesting CGP on tumor specimens. Until the results of prospective trials are reported, it will be difficult to curb the runaway train that is the routine clinical use of CGP. However, given the available data we recommend against routine CGP outside the context of a prospective precision medicine program or well-designed clinical trials.

## Abbreviations

CGP: Comprehensive genomic profiling
CML: Chronic myeloid leukemia
CRGA: Clinically relevant genetic alterations
EGFR: Epidermal growth factor receptor
GDT: Genotype directed therapy
HER2: Human epidermal growth factor 2
MAPK: Mitogen-activated protein kinase
PI3K: Phosphatidylinositol 3-Kinase
